# HINN: Hierarchical Input Neural Network identifies multi-omics biomarker for cognitive decline

**DOI:** 10.21203/rs.3.rs-7576397/v1

**Published:** 2025-09-17

**Authors:** Yashu Vashishath, Sarah Beaver, Fahad Saeed, Serdar Bozdag

**Affiliations:** 1 Department of Computer Science and Engineering, University of North Texas, Denton, TX, USA.; 2 BioDiscovery Institute, University of North Texas, Denton, TX, USA.; 3 Center of Computational Life Sciences, University of North Texas, Denton, TX, USA.; 4 Department of Mathematics, University of North Texas, Denton, TX, USA.; 5 School of Computing and Information Sciences, Florida International University, Miami, FL, USA.

**Keywords:** Cognitive decline, Alzheimer’s disease, multi-omics, cognitive scores, data integration, biologically-related feature selection, GWAS, machine learning, deep neural network

## Abstract

Understanding complex diseases requires models that can integrate diverse layers of biological data while yielding insights that are biologically interpretable. Although multi-omics integration with machine learning (ML) has advanced disease prediction and biomarker discovery, most existing approaches overlook the hierarchical and regulatory relationships that connect these molecular layers. Here, we present the Hierarchical Input Neural Network (HINN), a deep learning framework that incorporates known cross-omics relationships directly into its architecture, capturing the flow of information from genomics to epigenomics, transcriptomics, and downstream biological processes. By embedding these relationships, HINN improves both predictive performance and biological interpretability.

We applied HINN to blood-derived multi-omics data from individuals with Alzheimer’s disease or mild cognitive impairment to predict cognitive scores from standardized assessments. HINN outperformed both baseline and state-of-the-art models and pinpointed multi-omics biomarkers—including SNPs and promoter-region CpG sites in *ATP6V1C1* and *RCHY1* —that were significantly correlated with plasma p-Tau181 levels. These features map to biologically relevant processes with potential implications for cognitive decline.

Our findings demonstrate how combining deep learning with biological knowledge can uncover interpretable, blood-based biomarkers for cognitive decline due to complex diseases such as Alzheimer’s. All code and data are openly available at https://github.com/bozdaglab/HINN.

## 1 Introduction

Understanding and predicting diseases remains a critical challenge in healthcare. In recent years, scientists have increasingly turned to multi-omics data and machine learning (ML) approaches to unravel complex biological systems. Each omics dataset offers unique insights, but their integration provides a comprehensive view of disease mechanisms [1]. ML models excel at identifying hidden patterns within high-dimensional data that traditional statistical methods might overlook.

The potential of multi-omics data integration is particularly promising in disease prediction. ML algorithms have demonstrated remarkable capabilities in improving early disease detection, identifying biomarkers for rare conditions, and developing personalized treatment strategies [2, 3]. By combining diverse biological datasets, researchers can generate more precise predictions of disease susceptibility, progression, and potential treatment responses.

Deep Neural Networks (DNN) have emerged as a powerful approach for integrating biological data [4]. Notably, researchers have developed innovative methods to incorporate biological pathway knowledge directly into neural network architectures [5]. For example, Deng et al. [6] created a novel approach called pathway-guided deep neural network (PGNN) that integrates pathway information into DNN, improving both the interpretability of the model and drug sensitivity prediction. Their work demonstrated how computational models could reflect biological mechanisms, such as tracking how drugs interact with disease-related pathways. This approach was used in another method, called pathway-aware multilayered hierarchical network (P-NET) [7]. The model offers enhanced interpretability by incorporating known biological relationships across different omics layers, effectively illustrating the flow of information between them. P-NET incorporates prior biological knowledge to connect genes to the pathways they belong to, forming what are referred to as customized connections. By leveraging these customized connections, P-NET captures complex interactions among genes, pathways, and higher-order biological processes.

Despite these advances, existing multi-omics ML methods have significant limitations. Most models fail to comprehensively capture the hierarchical and regulatory relationships between different omics layers. For example, DNA methylation can suppress or activate gene expression [8], and single nucleotide polymorphisms (SNPs) can influence both methylation and transcriptional activity [9]. These cross-layer regulatory effects are biologically grounded, yet are often ignored in existing models, which treat omics layers independently. Moreover, current approaches often lack flexibility to incorporate biological knowledge and regulatory relationships between omics during feature selection [10].

**Figure 1:**
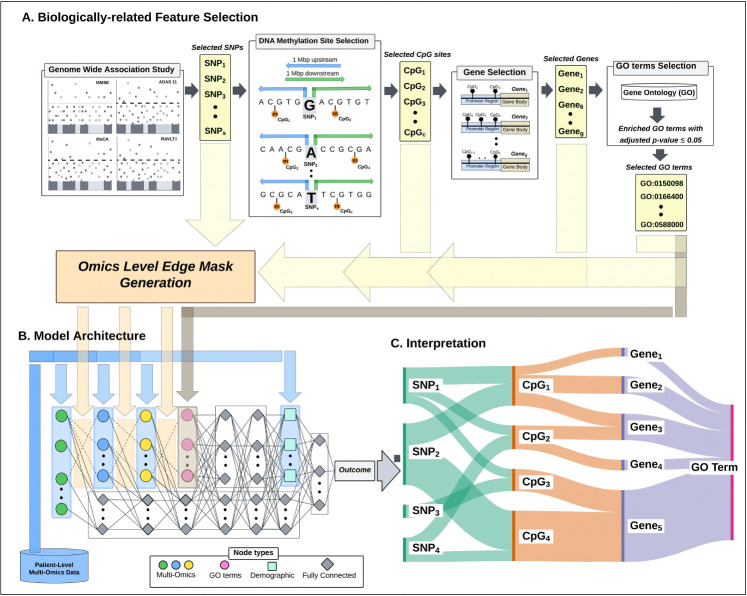
Overview of the Hierarchical Input Neural Network (HINN) framework. (A) The biologically informed feature selection process begins by identifying significant SNPs via genome-wide association studies (GWAS). CpG sites located within a ±1 Mb window around the significant SNPs and overlapping promoter regions are selected. Genes linked to these CpG sites are then retained, and their associated Gene Ontology (GO) terms are determined through enrichment analysis. (B) HINN integrates multi-omics data—SNPs, DNA methylation, and gene expression—using a hierarchical architecture. Each data modality is represented as a distinct input layer, with biologically meaningful interconnections across layers (see Figure S3A for architectural details). Demographic information is incorporated into the model at later stages, enhancing the predictive context with patient-level variables.(C) The model’s interpretability is enhanced by visualizing key predictive features across omics layers, allowing biological insight into decision-making.

To address these challenges, we propose a novel deep learning framework: the Hierarchical Input Neural Network (HINN) (Figure 1). HINN captures biologically plausible information flow by embedding known regulatory hierarchies—linking SNPs to CpG methylation sites to gene expression—directly into the network’s architecture. This biologically structured input design enhances interpretability while improving predictive accuracy incorporating diverse omics types.

As a case study, we applied HINN to blood-derived multi-omics data to predict cognitive function, focusing on standardized assessments such as the Mini-Mental State Examination (MMSE) [11], Montreal Cognitive Assessment (MoCA) [12], Alzheimer’s Disease Assessment Scale–Cognitive Subscale 11 tasks (ADAS11) [13], and Rey Auditory Verbal Learning Test (RAVLT.immediate) [14]. These assessments remain widely used for diagnosing neurodegenerative conditions like Alzheimer’s disease (AD) and mild cognitive impairment (MCI), but their clinical interpretation is often subjective and limited in sensitivity [15, 16].

Our results demonstrate that HINN not only improves the prediction of cognitive performance from blood-based multi-omics data but also enables biologically grounded interpretation. Specifically, HINN uncovers biomarkers consisting of SNPs, CpG sites and genes that are correlated to plasma p-Tau181, a clinically validated biomarker of AD pathology. These cascades highlight potential genes involved in lysosomal function, protein breakdown, and lipid metabolism—key biological processes linked to cognitive health and neurodegeneration. This integrative framework bridges deep learning and systems biology to support interpretable, blood-based biomarker discovery for cognitive function. All code, sample data, and instructions are publicly available at https://github.com/bozdaglab/HINN under the Creative Commons Attribution–NonCommercial 4.0 International License.

## 2 Results

### 2.1 Biologically Related Feature Selection and Architecture of HINN

To model molecular determinants of cognitive function, we implemented a biologically guided strategy to integrate SNPs, DNA methylation, and gene expression. Genome-wide association studies (GWAS) identified 297 SNPs associated with all four cognitive assessment tests (i.e., MMSE, MoCA, ADAS11, and RAVLT.Immediate) (adjusted *p* ≤ 0.01; Figure S1). To find DNA methylation sites associated with these SNPs, CpG sites located within ±1 Mb of them were selected, yielding 13,688 DNA methylation sites. Gene expression probes were included if their associated genes contained at least one selected CpG site in the promoter region, resulting in 1,727 probes. To identify biological processes (BP) associated with these genes, we performed an enrichment analysis and obtained 158 GO BP terms (adjusted p-value ≤ 0.05). The features without any cross-layer biological relationship were removed reducing the SNP features to 254, which appear on all chromosomes with the highest frequency on chromosome 8 (Figure S2. This filtering step produced a layered multi-omic feature set with only biologically connected features retained.

To leverage the directional flow of regulatory information, we developed the Hierarchical Input Neural Network (HINN), a deep learning framework that mirrors known biological relationships: from genetic variants to epigenetic regulation, transcriptomic output, and functional annotation via GO terms (Figure S3A). Each omic layer is structurally constrained by prior biological relationships, preserving information flow through sparse, biologically meaningful connections. To maintain information flow, each layer from CpG onward also includes 20 additional fully connected perceptrons [17]. After omics integration layer, HINN has four dense layers and demographic integration layer, which incorporates demographic features (i.e., age, sex, education, APOE4, and plasma p-Tau181) before computing its prediction.

### 2.2 Cognitive Score Prediction and Performance Comparison

HINN was applied to predict cognitive performance across four standard assessments: MMSE, MoCA, ADAS11, and RAVLT.Immediate. Table S1 summarizes the predictive performance using mean absolute error (MAE) and mean squared error (MSE). MAEs for MMSE (2.07) and MoCA (3.00) were low, indicating strong predictive accuracy. Slightly higher errors for ADAS11 (4.54) and RAVLT.Immediate (9.10) reflect their broader scoring ranges (0–70 and 0–75, respectively).

We compared HINN to a set of baseline and advanced models: Support Vector Machine (SVM), Random Forest (RF), Deep Neural Network (NN), and L1-regularized Logistic Regression (LR). Additionally, we implemented a Pathway-Guided Neural Network (PGNN), drawing on the architectures proposed in previous biologically informed models [6, 7], as a benchmark for interpretability and biological relevance. Since, unlike HINN, PGNN does not explicitly model hierarchical interactions between omics layers, multi-omic features—SNPs, DNA methylation, and gene expression—were concatenated into a single input layer which sparsely connected to a hidden layer based on shared GO term annotations (Figure S3B).

**Figure 2:**
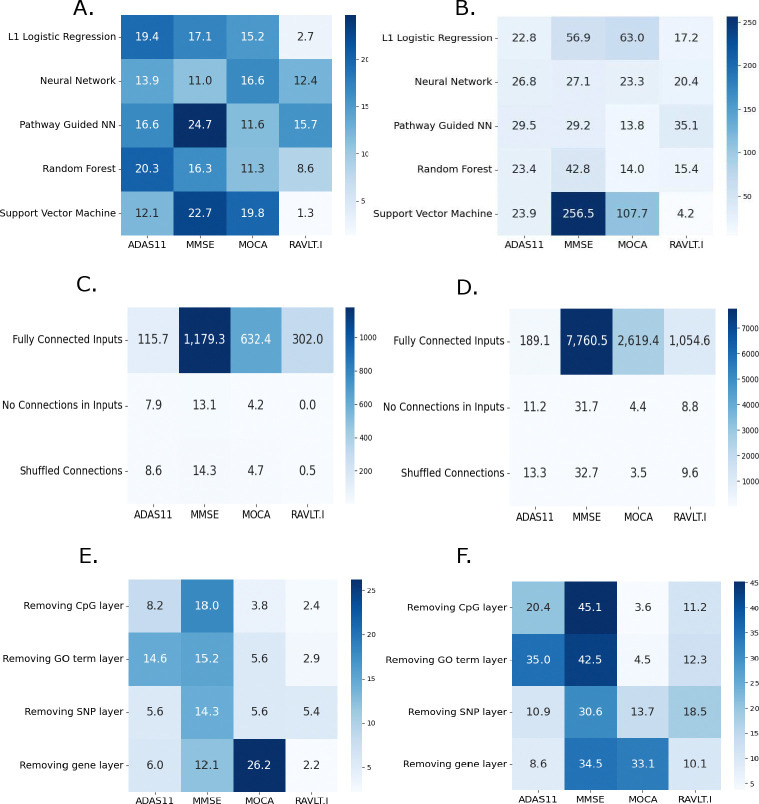
Percent improvement of HINN’s predictions over other models is evaluated by comparing the mean of error across three runs: (A) MAE and (B) MSE comparisons across cognitive tests, showing HINN’s improved performance. (C) MAE and (D) MSE demonstrating the benefit of biologically-related sparse connections between network layers. (E) MAE and (F) MSE illustrating the contribution of each omic input layer and GO term based connections to prediction performance.

Across all cognitive assessments, HINN consistently outperformed other baseline models(Figure 2A–B). In particular, the performance gains over PGNN highlight the added value of encoding inter-layer regulatory flow rather than relying solely on feature-level biological grouping. These results support the utility of HINN’s structured architecture in predictive accuracy.

### 2.3 Biological Layer Connections Enhance Model Accuracy

To evaluate the impact of biologically structured input layers in HINN, we conducted ablation studies targeting both inter-layer connectivity and individual omics modalities. These experiments isolate the architectural contributions that drive predictive performance.

#### 2.3.1 Impact of Inter-Layer Connectivity

We tested three alternative connectivity schemes to assess the importance of HINN’s biologically constrained structure. In the first variant, all nodes between adjacent layers were fully connected, effectively removing curated SNP–CpG site–gene–GO mappings and allowing unrestricted flow of information across layers. In the second variant, all inter-omic-layer connections were eliminated, removing biologically curated inter-layer connections but preserved the hierarchical sequence of omics layers. Specifically, outputs from each omics input were passed through a dense layer, and the result was concatenated with the next omics layer before being processed further. This design preserved directional flow across omics levels but eliminated biological specificity in the connections, simulating a structure-aware but biologically agnostic network. The third variant preserved the original number of inter-layer connections but randomized their pairings, disrupting the biological relationships among omic layers while maintaining architectural sparsity and integration methodology.

All alternative configurations led to substantial degradation in performance, with MSE ranging from 3.5% to 7,760.5% higher compared to the original HINN model (Figure 2C–D). These results demonstrate that preserving biologically meaningful inter-layer connections is critical for effective multi-omic integration and cognitive score prediction.

#### 2.3.2 Contribution of Input Modalities

To assess the influence of individual omics input layers, we systematically removed one modality at a time while keeping the rest of the architecture unchanged. We also tested a version of HINN without the GO term layer, allowing the gene expression layer to connect directly to the dense layers.

Removing any omics input layer led to an increase in the prediction error of cognitive tests up to 45% (Figure 2E–F). For instance, removal of the gene layer resulted increased prediction error for MOCA by 26.2% and 33.1% in MAE and MSE, respectively highlighting the critical role of gene-level features in cognitive prediction. Similarly, substantial increase in prediction error were observed upon removal of the GO term layer, particularly for ADAS11 and MMSE, where HINN achieved 35.0% and 42.5% improvement in MSE, respectively. These findings highlight the complementary value of each omics type—genetic, epigenetic, and transcriptomic—in capturing regulatory signals relevant to cognitive function.

### 2.4 Model Interpretation and Multi-Omic Associations with Cognitive Function

To interpret the molecular drivers of cognitive function captured by HINN, we applied the DeepLIFT algorithm [18], implemented via PyTorch and integrated with TensorFlow 3.7, to quantify the contribution of SNPs, CpG sites, and gene expression probes across different cognitive scores. This gradient-based method measures how small perturbations in input features influence model predictions, enabling transparent interpretation of complex neural networks.

**Figure 3:**
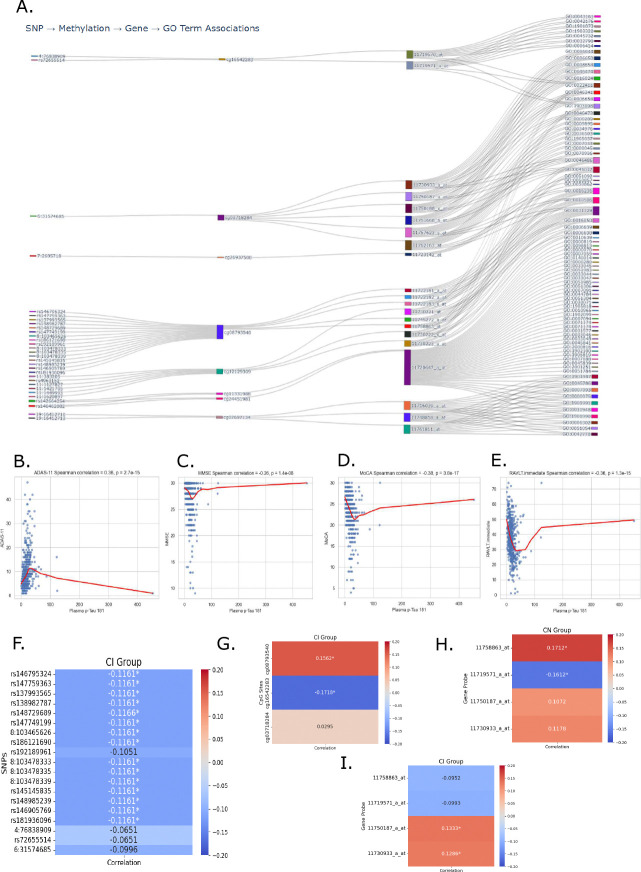
Multimodal associations linking DNA methylation, gene expression, and cognitive biomarkers. (A) Sankey diagram illustrating gene-level integration of top-ranked SNPs, CpG sites, and gene expression probes selected by the HINN model. The visualization highlights convergence of features across omics layers onto shared SNPs, methylation probes, gene probes, leading to connected GO terms. (**B–E**) Spearman correlations between plasma p-Tau181 levels and cognitive performance measures: (B) ADAS-11, (C) MMSE, (D) MoCA, and (E) RAVLT.immediate. Higher p-Tau levels were consistently associated with worse performance across cognitive domains. (F-I) Spearman correlation between identified features in (A) and plasma p-Tau181 levels for CN or CI patients. Several features had significant correlation with p-Tau181, suggesting a link between identified features and a known biomarker of cognitive function.

Feature importance scores were averaged across models trained on different cognitive tests to identify consistently informative markers. While gene expression features exhibited more test-specific patterns of importance, SNP and DNA methylation features stood out for their consistent rankings in high percentile across all tests (Figure S4). To explore how these multi-omic signals converge within GO terms, we generated a Sankey diagram linking prioritized SNPs, CpG sites, and expression probes to downstream GO terms (Figure 3A). Each vertical block in the diagram represents one molecular layer, and the connecting flows show features that are jointly prioritized by HINN and meet the defined spatial–functional constraints. This structure allows us to see where genetic variants are located near methylation sites, which in turn are positioned in promoter regions of genes whose expression levels are associated with cognitive score. Mapping these genes to GO terms highlights the biological processes in which they participate, revealing clusters of features that point toward shared functional themes. While the diagram does not imply directionality or causation, it provides a visual summary of how genetic, epigenetic, and transcriptomic signals converge on related biological pathways.

The genes representing the probes, CpG sites and SNPs in Figure 3A - namely, *AGPAT1*, *ATP6V1C1*, *BUB1B*, *CARD11*, *C19orf62*, *PNPLA2*, *RCHY1* and *RNF5 -* have not been extensively studied in the context of cognitive decline. However, these genes may influence brain function indirectly through roles in endolysosomal acidification, intracellular trafficking, phospholipid biosynthesis, protein ubiquitination, and lipid metabolism, as identified by the GO terms associated with the gene probes.

To further analyze the association of the selected features to cognitive function, we analyzed the correlation between gene expression and CpG sites in two groups of individuals in ADNI: those with cognitively normal (CN) diagnosis, which included 157 individuals, and those with cognitive impairment (CI), comprising 298 individuals diagnosed with MCI or AD. Our analysis revealed a significant negative Spearman correlation between *PNPLA2* gene expression (probe 11730222_x_at & 11730223_a_at) and DNA methylation at probe cg12129309 in CI patients (Table 1). This indicates that hypomethylation of probe cg12129309 is associated with increased *PNPLA2* expression in individuals with CI. We also found similar correlation of CpG site cg03718284 and gene probe 11757623_s_at (*RNF5*) among CI individuals. We also observed significant relationships between CpG sites and gene expression probes among CN individuals. For instance, CpG site cg03718284 had significant positive correlation with probe 11762163_at (*RNF5*), showing that same CpG site can have different correlation based on disease state on the same gene. Few other CpG sites and gene probes had significant correlation as shown in Table 1 for CN individuals.

### 2.5 Correlation Between Gene Expression and Promoter Region DNA Methylation in CN and CI Patients Using ROSMAP Data

To evaluate the reproducibility of significant DNA methylation–gene expression associations identified in the ADNI dataset, we conducted the same analysis using the ROSMAP cohort. Because the exact CpG sites from ADNI were not profiled in ROSMAP, we instead examined CpG sites located within the promoter regions (annotated as TSS200 or TSS1500) of genes previously implicated in ADNI. Correlations between promoter methylation and gene expression were assessed separately for CN and CI individuals. Only CpG site and gene probe pairs demonstrating statistically significant associations (p ≤ 0.05) in at least one group are shown in Table 2.

In ADNI, we observed significant negative correlations between methylation at cg12129309 and expression of the *PNPLA2* gene in CI individuals, while no such relationship was seen in the CN group. In ROSMAP, two of three promoter CpG sites (cg13833525 and cg19713609) also showed significant negative correlations with gene expression in CI individuals, partially reproducing the ADNI findings. The third site (cg02568531) showed positive correlation between DNA methylation and gene expression for CI individuals which was not seen in ADNI cohort for any CpG site in promoter region of *PNPLA2* gene. The second site (cg19713609) also exhibited a significant positive correlation in CN individuals (*ρ* = 0.176, p = 0.035), a pattern not observed in ADNI either. This bidirectional relationship across cognitive groups in ROSMAP suggests potential context-specific epigenetic regulation of *PNPLA2*, possibly influenced by cognitive status or disease progression.

In ADNI, we identified a significant negative correlation between CpG methylation (cg03718284) and *RNF5* expression in CI individuals. In ROSMAP, we observed broad and consistent negative correlations between multiple promoter CpG sites and *RNF5* expression in both CN and CI groups. While the strength and number of significant correlations were greater in CN, the directionality was preserved across cognitive groups, reinforcing the robustness of this methylation–expression relationship.

In the ROSMAP cohort, we observed a consistent positive correlation between CpG methylation at sites cg13763617, cg08049198, and cg01466825 and *AGPAT1* expression in CN individuals, similar to findings from the ADNI dataset. We identified that cg02260340 had a negative correlation with *AGPAT1* expression in CI individuals, a pattern absent in ADNI dataset. Additionally, a significant negative correlation between CpG site cg14771938 and *AGPAT1* expression was identified in CN individuals within ROSMAP, previously not seen in ADNI cohort. In contrast, *ATP6V1C1* expression did not show significant correlations in either group in ADNI, however, ROSMAP results showed negative correlations in CN individuals (cg24478696, *ρ* = −0.177, p = 0.034; cg20490199, *ρ* = −0.187, p = 0.025), with no significant associations in CI participants.

We observed a cross-cohort parallel in disease-dependent epigenetic regulation between *RNF5* and *PNPLA2*. In ADNI, *RNF5* exhibited a bidirectional correlation — positive in CN and negative in CI — whereas in ROSMAP, a similar pattern emerged for *PNPLA2*, with a positive correlation in CN and negative in CI. This mirroring suggests a broader phenomenon in which cognitive impairment is associated with directional shifts in methylation–expression coupling, possibly reflecting shared mechanisms of epigenetic reprogramming in disease progression.

### 2.6 Tau-Association with Cognitive Decline

To link the prioritized multi-omic features with functional cognitive outcomes, we first evaluated the relationship between plasma p-Tau181 levels and cognitive performance. Spearman correlation analysis revealed that elevated concentrations of p-Tau181 were significantly associated with poorer scores across multiple domains: MMSE (*ρ* = −0.26, *p* = 1.35 × 10^−8^), MoCA (*ρ* = −0.38, *p* = 3.76×10^−17^), ADAS-11 (*ρ* = 0.36, *p* = 2.67 × 10^−15^), and RAVLT.immediate (*ρ* = −0.36, *p* = 1.34 × 10^−15^) (Figure 3B–E). These associations reinforce the utility of this biomarker as a surrogate indicator of cognitive function.

To assess the biological relevance of model-prioritized features, we examined their associations with the same plasma marker [19]. Among features highlighted in the Sankey diagram, 16 SNPs showed significant negative correlation in CI individuals (Figure 3F). Notably, 15 of these variants were linked to a single CpG site, cg08793540, which itself showed significant positive correlation with the marker levels in the CI group (Figure 3G). A second site, cg16542283, also showed a significant negative correlation in the same population.

To further explore transcriptional regulation of p-Tau181, we evaluated model-prioritized gene expression features. The expression of *ATP6V1C1* via probe 11758863_at was positively correlated with p-Tau181 in CN individuals (Figure 3H), while probe 11719571_a_at (associated with *RCHY1*) was negatively correlated in the same cohort. Both cg08793540 and cg16542283 are located within promoter regions of their respective genes (*ATP6V1C1* and *RCHY1*), suggesting possible epigenetic regulation. The GO terms assoicated with probe 11758863 at of the gene *ATP6V1C1*-GO:0031329, GO:0016050, GO:0016236, and GO:0010506-are all directly or indirectly related to autophagy, a cellular process implicated in the clearance of P-Tau. In contrast, the GO terms linked to probe 11719571_a_at of gene *RCHY1* (like GO:0043161, GO:0042176, GO:1901873, GO:1903320, GO:0045732, GO:0006414, GO:1903008, GO:0032790, GO:0022411) are unified by their association with cellular homeostasis, which is particularly vital in the prevention of cognitive decline.

In CI individuals, two probes for *AGPAT1* (11750187_a_at and 11730933_a_at) showed significant positive correlations with p-Tau181 (Figure 3I), implicating this gene in tau-related pathology as well. Together, these results illustrate how genetic and epigenetic regulation may converge on gene expression patterns linked to cognitive function.

## 3 Discussion

In this study, we developed a biologically structured deep learning framework, the Hierarchical Input Neural Network (HINN), to integrate blood-derived multi-omics data for predicting cognitive function. By mirroring the regulatory hierarchy from genetic variation to epigenetic modification and transcriptional activity, HINN enables interpretable and accurate predictions of cognitive test outcomes. Our findings demonstrate that explicitly modeling molecular dependencies—rather than treating each omics layer independently—improves predictive accuracy while preserving biological interpretability.

A key contribution of this work is the identification of multi-omic regulatory biomarkers linking genomic variants (SNPs), DNA methylation sites, and gene expression that are significantly correlated with plasma p-Tau181 levels, a clinically validated marker of tau pathology and cognitive decline. These biomarkers meet stringent spatial and functional constraints: the SNP lies within 1 Mb of the associated DNA methylation site, and the DNA methylation site is located in the promoter region of the corresponding gene. This arrangement raises the hypothesis of a potential regulatory cascade in which upstream genetic variation could influence DNA methylation status, thereby modulating transcriptional activity and ultimately affecting tau-related pathology. Nonetheless, this interpretation is speculative and will require further biological analyses and experimental validation to determine its validity.

To evaluate the functional relevance of these biomarkers, we examined associations with CpG sites and expression probes for genes such as *ATP6V1C1*, *RCHY1*, and *AGPAT1*. These features, fulfilling the defined positional–regulatory criteria, were significantly associated with p-Tau181 levels and mapped to regulatory regions, suggesting a plausible epigenetically mediated mechanism of action. Taken together, our findings suggest that the features highlighted by HINN are not just statistically significant—they also fit into coherent biological patterns that make sense given what is known about molecular regulation. The fact that our model-identified relationships align with established and plausible mechanisms, adds weight to the idea that cognitive function and related diseases are shaped by interconnected processes across genetic, epigenetic, and transcriptional layers. Interpretable deep learning offers a way to reveal these complex, multi-layer systems in a systematic and biologically meaningful way.

While HINN’s design captures structured relationships across genetic, epigenetic, and transcriptomic layers, it does not yet incorporate feedback loops, post-transcriptional regulation, or protein-level interactions. Future extensions could integrate proteomic or metabolomic data, and the use of longitudinal cohorts would enable the modeling of temporal changes in molecular profiles alongside cognitive outcomes. In addition, tuning the hyperparameters of the model could further enhance predictive performance and improve the prioritization of biologically meaningful features.

Overall, HINN offers a biologically interpretable and statistically robust framework for multi-omics integration. By identifying sets of SNPs, DNA methylation sites, and gene expression features that are spatially and functionally connected and significantly associated with cognition-related biomarkers, the approach provides a foundation for generating targeted hypotheses and guiding follow-up experimental studies into the molecular basis of brain health.

## 4 Methods

### 4.1 Dataset

We utilized data from the Alzheimer’s Disease Neuroimaging Initiative (ADNI) (adni.loni.usc.edu), a longitudinal study launched in 2003 to investigate biomarkers associated with MCI and the progression of AD [20, 21]. Our study incorporated cognitive assessments (MMSE, MoCA, ADAS11, and RAVLT.Immediate), whole genome sequencing (WGS), DNA methylation, and gene expression data from 736, 704, 576, and 744 participants, respectively. In addition, we included demographic variables (i.e., gender, race, age at blood donation, and education), APOE4 genotype, and levels of plasma p-Tau181 for 736 participants. Samples with missing data in any modality were excluded to minimize confounding effects, leaving total of 455 samples with all data modalities.

We also obtained 492 individuals with DNA methylation and 728 individuals with gene expression data from the ROSMAP dataset to supplement our analysis [22].

### 4.2 Patient Characteristics

The processed ADNI dataset included 455 participants with complete data across all modalities. Of these, 157 were classified as CN and 298 were classified as CI (222 MCI and 76 AD cases). Participants’ ages ranged from 55 to 93 years (mean: 75). Blood p-tau protein levels varied between 0.36 and 451.39 pg/mL (mean: 20.05). The cohort comprised 261 males and 194 females, with the majority identified as White (n = 446), while other racial groups included Black (n = 2), more than one race (n = 2), and American Indian/Alaskan (n = 5). APOE4 genotype distribution included 261 individuals with no copies of the APOE4 allele, 160 with only one copy of the APOE4 allele (i.e., heterozygous), and 34 with two copies of the APOE4 allele (i.e., homozygous, *ϵ*4/*ϵ*4).

Additionally, the ROSMAP dataset had 285 participants who had both DNA methylation and gene expression data, which included 143 CN (score = 1) and 142 CI (score = 4–6) participants based on post-mortem consensus cognitive diagnosis. The participants ranged in age from 66 to 90+ years at the time of death, with a mean age of 86. The cohort included 110 males and 175 females, with an approximately equal distribution between CN and CI individuals. All participants were of single race.

### 4.3 Genotype Data Processing

Genome-wide association study (GWAS) analysis was conducted using PLINK v1.06 [23]. Following established protocols [24], SNPs and samples with a missing call rate ^>^ 95% were excluded. SNPs with a minor allele frequency (MAF) < 5% or a Hardy-Weinberg equilibrium test p-value > 10^−6^ were also removed.

A linear regression model was used to assess associations between SNPs and cognitive measurements, resulting in four separate GWAS analyses. Across these analyses, 297 common SNPs were identified (Bonferroni-adjusted p-value < 10^−8^). To ensure relevance to epigenetic regulation, SNPs without a corresponding methylation site within 1 MB upstream or downstream of a gene promoter region were excluded. This filtering step reduced the final set of SNPs used in downstream analyses to 254.

### 4.4 DNA Methylation Data Processing

The ADNI DNA methylation profiling was performed using the Illumina Infinium HumanMethylationEPIC BeadChip Array [25] at basedline, +1 year and +2 year visits of the individuals. The dataset consisted of 1,920 samples, including 1,719 unique samples and 201 technical replicates from 653 individuals. For this study, we utilized the DNA methylation profile from the most recent visit.

Data processing was conducted using the Minfi [26] package in R/Bioconductor [27], starting with raw intensity data from .idat files (*.Red.idat* and *.Grn.idat*). The raw data were loaded using *read.metharray.exp()* function to generate a rgSet object, from which detection p-values were calculated to assess the data quality. Samples with an average detection p-value > 0.05 were excluded, and probes failing in any sample or containing SNPs at CpG sites were removed. The remaining samples underwent quantile normalization using *preprocessQuantile()* function, resulting in a final dataset of approximately 684,000 probes. From this processed data, beta values (*β*-values) were computed using *getBeta()* function, which represent the ratio of methylated probe intensity (M) to the total signal intensity (i.e., sum of methylated and unmethylated (U)) (Equation 1). A total of 13,688 CpG sites located within 1 Mb upstream or downstream of identified significant SNPs and in promoter region of genes, were used in downstream analysis where the window size of 1 Mb was determined based on a previous study [28].

(1) $$\beta =\frac{M}{M\;+\;U}$$

ROSMAP DNA methylation beta values were extracted for CpG sites located in the 200bp and 1500bp upstream of transcription start site (i.e., TSS200 and TSS1500) of the genes. DNA methylation data from ROSMAP study did not require any processing as the data obtained was already normalized.

### 4.5 Gene Expression Data Processing

ADNI gene expression data was processed at the probe level to preserve signal specificity. Control probes and samples with an RNA Integrity Number below 6.9 were excluded [29]. We identified 1,727 probes corresponding to genes with at least one of the 13,688 identified CpG sites in their promoter region. Prior to further analysis, expression data was min-max normalized.

For functional enrichment analysis, these 1,727 genes were mapped to biological process GO terms. A total of 158 GO terms were identified as significantly enriched (p-value < 0.05) for this gene set.

Gene expression data obtained from ROSMAP study was already normalized therefore, we used the data without any processing steps.

### 4.6 Mathematical Foundation of the HINN Architecture

To formalize the biological relationships in our hierarchical network, we developed a mathematical framework that governs feature interactions across different omics layers. The core of our approach lies in creating structured connections between layers that reflect known biological dependencies. Figure S3A shows the flow of our model with details regarding each layer.

#### 4.6.1 Mask-Based Connection Framework

To enforce biologically meaningful connections between omics layers, we employ a mask-based connection framework. This mechanism uses a binary mask matrix $M_{i}$ for each layer i to specify permitted interactions between the input and output features of a given layer.

Let the input to the layer l be denoted as $X_{l}\in R^{c\times d}$, where c is the number of samples and d is the number of input features. The corresponding output is represented as $Y_{l}\in R^{c\times e}$, where e denotes the output feature dimension. The transformation is parameterized by a weight matrix for the initial layer $W_{l}\in R^{d\times e}$ and a bias vector $B_{l}\in R^{e}$.

The transformation from one layer to the next is defined by the masked linear mapping:

(2) $$f\left(X_{l},M_{l}\right)=\sigma \left(X_{l}\cdot \left(M_{l}⊙W_{l}\right)+B_{l}\right)$$

In Equation 2, ⊙ denotes element-wise (Hadamard) multiplication, which ensures that only connections allowed by the mask $M_{l}$ are retained. The matrix product $X_{l}\cdot \left(M_{l}⊙W_{l}\right)$ computes the masked transformation of the input, followed by the addition of a bias term $B_{l}$ and a non-linear activation function σ(⋅) to model complex relationships.

The mask $M_{l}\in {\left\{0,\;1\right\}}^{d\times e}$ in layer l encodes known biological relationships (e.g., SNP–CpG, CpG–gene or gene-GO term links), thereby constraining the learning process to respect established molecular hierarchies.

For omics input layers beyond the first, we apply a feature-wise transformation to adjust individual features prior to integration with other omics layer. This is achieved through the identity-based mapping function $g\left(X_{l}\right)$ for l≥2, which serves as a preprocessing step before integrating with the biologically-guided transformation $f\left(X_{l-1},M_{l-1}\right)$:

(3) $$g\left(X_{l}\right)=X_{l}\cdot \left(I⊙W_{s}\right)$$

Here, $I\in R^{e\times e}$ is the identity matrix, and the element-wise multiplication $I⊙W_{l}$ restricts $W_{l}\in R^{e\times e}$ to its diagonal, enforcing a one-to-one correspondence between each input feature and its associated trainable weight in layer l. This operation preserves the dimensionality of the input and ensures that each input feature is assigned a separate trainable weight.

#### 4.6.2 Layer-Specific Implementations

The hierarchical propagation of biological signals is modeled based on whether inter-layer interactions are additive (amplifying) or decremental (attenuating). The piecewise definition is given by:

(4) $$Y_{l}=\left\{\begin{array}{ll} \sigma \left(\frac{g\left(X_{l}\right)}{f\left(Y_{l-1},\;M_{l-1}\right)}+B_{l}\right) & \;\text{if}\;\text{decremental}\;\\ \sigma \left(g\left(X_{l}\right)\cdot f\left(Y_{l-1},M_{l-1}\right)+B_{l}\right) & \;\text{if}\;\text{additive}\; \end{array}\right.$$

In Equation 4, l denotes the current layer. The function $g\left(X_{l}\right)$ transforms the input of the current layer, while $f\left(Y_{l-1},M_{l-1}\right)$ incorporates the modulated output from the previous layer using mask $M_{l-1}$. This formulation enables the model to flexibly represent biologically meaningful interactions.

The following layer-specific implementations reflect known biological mechanisms used in our model:

##### SNP Layer (Foundation):

The initial layer transforms genetic variants ($X_{1}$) using connectivity masks that reflect genomic relationships, and the output of the first layer is calculated in the same dimensions as that of the second layer using Equation 5.

(5) $$Y_{1}=f\left(X_{1},M_{1}\right)$$

##### DNA Methylation Layer:

Epigenetic information ($X_{2}$) is integrated with upstream genetic influences through multiplicative interactions to generate output of the second layer after activation using the non-linear activation function as depicted in Equation 6.

(6) $$Y_{2}=\sigma \left(\left(g\left(X_{2}\right)⊙Y_{1}\right)+B_{2}\right)$$

##### Gene Expression Layer:

Transcriptomic data ($X_{3}$) incorporates the inhibitory effects of DNA methylation through a division-based transformation as shown in Equation 7.

(7) $$Y_{3}=\sigma \left(g\left(X_{3}\right)/f\left(Y_{2},M_{2}\right)+B_{3}\right)$$

##### GO Term Layer:

There was no new input in GO term layer but the connections between gene expression layer and this layer were calculated based on Equation 8.

(8) $$Y_{4}=f\left(Y_{3},M_{3}\right)$$

#### 4.6.3 Fully Connected Component of HINN

To mitigate potential information loss caused by sparse connections between omics input layers, we introduced additional nodes in the DNA methylation, gene expression, and GO term layers that are fully connected to their preceding layers. First, a 20-node dense layer was added to the DNA methylation layer connected to the SNP layer to capture and forward all relevant information from the genetic features. These nodes, along with the output $Y_{2}$ (Equation 6), were fully connected to a second set of 20 dense nodes in the gene expression layer. This second set, together with $Y_{3}$ (Equation 7), was then fully connected to a third dense layer with 20 nodes in the GO term layer.

The output of the GO term layer and the dense nodes were subsequently passed through four dense layers with 128 nodes each, structured as a sequence of batch normalization (momentum = 0.9, epsilon = 0.005), dense, and dropout layers (rate = 0.7) for stability and regularization. Each dense layer employed ReLU activation and L2 regularization, with variance scaling used for weight initialization. The resulting representation of multi-omics input was passed through a dense layer of 20 nodes. The output of which was then concatenated with six demographic and biomarker features—APOE4 status, p-Tau181 levels, age, gender, education, and race—forming a joint feature vector that integrates both molecular and clinical information. The new combined representation was fed into one additional set of batch normalization, dense (128 nodes), and dropout layers, before being passed to a final output node with a linear activation function to predict the cognitive score.

The dataset was divided into 70% training, 30% testing, and 20% of the training data was used as validation for early stopping and restoring the best parameters. The model was executed three times to compute mean and standard deviation of evaluation metrics.

### 4.7 Baseline machine learning models

To compare HINN’s predictive performance, we used several baseline methods namely, L1, SVM, RF, NN, and PGNN. To ensure a fair comparison, we divided the whole data into 70% training data and 30% testing data, and used the same train and test split for all the models. To tune the hyperparameters, we used 10-fold-cross validation using the training data. The list of hyperparameters for each baseline model is shown in Table S2.

PGNN architecture was built by combining multi-omics input to a dense layer with 158 nodes based on connections between GO terms and omics data while keeping rest of the architecture same as that of HINN (Figure S3B). All parameters for different layers were kept consistent with HINN to have a fair comparison between HINN and PGNN. All models were also executed three times for a consistent comparison.

## Supplementary Material

Supplement 1

## Figures and Tables

**Table 1:** Correlation between gene expression probes and CpG sites for cognitively normal and impaired individuals in ADNI.

DNA Methylation Site	Gene Expression Probe	Gene Symbol	Cognitively Normal		Cognitively Impaired	
		
			Corr.	P-Val.	Corr.	P-Val.

cg03718284	11730933_a_at	AGPAT1	**0.195**	**0.014**	−0.067	0.248
cg03718284	11750187_a_at	AGPAT1	0.039	0.628	−0.011	0.847
cg03718284	11750188_x_at	AGPAT1	0.089	0.269	−0.083	0.153
cg03718284	11751668_a_at	AGPAT1	0.090	0.262	−0.048	0.411
cg08793540	11722181_a_at	ATP6V1C1	−0.147	0.066	−0.053	0.360
cg08793540	11722182_a_at	ATP6V1C1	0.016	0.847	−0.093	0.109
cg08793540	11722183_s_at	ATP6V1C1	−0.048	0.554	−0.068	0.244
cg08793540	11745272_a_at	ATP6V1C1	−0.087	0.280	−0.055	0.343
cg08793540	11758863_at	ATP6V1C1	−0.058	0.472	−0.031	0.598
cg11331988	11720647_a_at	BUB1B	0.047	0.562	−0.002	0.976
cg24451981	11720647_a_at	BUB1B	−0.025	0.759	−0.064	0.268
cg07697134	11716039_a_at	C19ORF62	0.107	0.183	−0.003	0.959
cg07697134	11748858_a_at	C19ORF62	0.038	0.634	−0.016	0.779
cg07697134	11761811_at	C19ORF62	**−0.224**	**0.005**	0.045	0.438
cg26937500	11723142_at	CARD11	**−0.194**	**0.015**	−0.012	0.830
cg12129309	11730221_at	PNPLA2	0.103	0.201	−0.053	0.359
cg12129309	11730222_x_at	PNPLA2	−0.075	0.347	**−0.163**	**0.005**
cg12129309	11730223_a_at	PNPLA2	−0.086	0.287	**−0.186**	**0.001**
cg16542283	11719570_at	RCHY1	−0.071	0.376	−0.002	0.969
cg16542283	11719571_a_at	RCHY1	−0.030	0.707	0.035	0.545
cg03718284	11757623_s_at	RNF5	−0.058	0.467	**−0.189**	**0.001**
cg03718284	11762163_at	RNF5	**0.280**	**0.000**	0.061	0.298

* *Statistically significant correlations are in bold (p-value* ≤ 0.05*).*

**Table 2:** Significant correlation between gene expression probes and CpG sites for CN and CI individuals in ROSMAP.

DNA Methylation Site	Gene Expression Probe	Gene Symbol	Cognitively Normal		Cognitively Impaired	
		
			Corr.	P-Val.	Corr.	P-Val.

cg14771938	1697790	AGPAT1	**−0.194**	**0.020**	−0.028	0.737
cg13763617	1697790	AGPAT1	**0.193**	**0.021**	−0.035	0.683
cg08049198	1697790	AGPAT1	**0.182**	**0.029**	−0.080	0.342
cg01466825	1697790	AGPAT1	**0.173**	**0.039**	−0.066	0.432
cg02260340	1697790	AGPAT1	−0.163	0.051	**−0.172**	**0.041**
cg24478696	5550750	ATP6V1C1	**−0.177**	**0.034**	−0.022	0.793
cg20490199	5550750	ATP6V1C1	**−0.187**	**0.025**	−0.112	0.185
cg19713609	1787923	PNPLA2	**0.176**	**0.035**	**−0.198**	**0.018**
cg02568531	1787923	PNPLA2	0.046	0.588	**0.209**	**0.012**
cg13833525	1787923	PNPLA2	−0.150	0.075	**−0.232**	**0.006**
cg18928683	2044927	RNF5	**−0.256**	**0.002**	−0.092	0.275
cg15982308	2044927	RNF5	**−0.247**	**0.003**	−0.112	0.183
cg09043226	2044927	RNF5	**−0.222**	**0.008**	−0.105	0.214
cg23464264	2044927	RNF5	**−0.218**	**0.009**	−0.131	0.121
cg18191873	2044927	RNF5	**−0.207**	**0.013**	−0.094	0.267
cg24534346	2044927	RNF5	**−0.197**	**0.018**	**−0.185**	**0.028**
cg25733934	2044927	RNF5	**−0.196**	**0.019**	−0.117	0.165
cg08450897	2044927	RNF5	**−0.196**	**0.019**	−0.071	0.400
cg08049198	2044927	RNF5	**−0.186**	**0.026**	**−0.211**	**0.012**
cg09301199	2044927	RNF5	**−0.177**	**0.034**	**−0.166**	**0.049**
cg11043450	2044927	RNF5	**−0.175**	**0.036**	−0.085	0.312
cg14771938	2044927	RNF5	**−0.173**	**0.039**	−0.162	0.054
cg03237964	2044927	RNF5	**−0.167**	**0.047**	**−0.167**	**0.047**
cg01466825	2044927	RNF5	−0.158	0.060	**−0.192**	**0.022**
cg13763617	2044927	RNF5	−0.110	0.192	**−0.244**	**0.003**
cg22673001	2044927	RNF5	−0.164	0.050	−0.062	0.461

* *Statistically significant correlations are in bold (p-value* ≤ 0.05*).*

## Data Availability

The datasets were derived from sources in the public domain: ADNI: https://adni.loni.usc.edu/. ROSMAP: https://www.synapse.org/Synapse:syn3219045
