# Supplementary material for: HINN: Hierarchical Input Neural Network identifies multi-omics biomarker for cognitive decline
Source: Res Sq. 2025 Sep 17:rs.3.rs-7576397. Preprint. [Version 1] doi: 10.21203/rs.3.rs-7576397/v1 (PMC12458612; doi:10.21203/rs.3.rs-7576397/v1)
Supplement: Supplement 1 [file NIHPPRS7576397v1-supplement-1.pdf]

8 Supplementary Tables and Figures

Table S1: MAE and MSE with standard deviation for prediction of cognitive test scores using HINN.

**Cognitive Test**	**MAE** (Mean ± SD)	**MSE** (Mean ± SD)	**Range of Actual Score**
ADAS11	4.54 ±0.21	51.42 ±8.85	0 (CN) to 70 (AD)
MMSE	2.07 ±0.18	9.07 ±1.81	0 (AD) to 30 (CN)
MoCA	3.00 ±0.12	18.66 ±1.04	0 (AD) to 30 (CN)
RAVLT.Immediate	9.10 ±0.17	130.20 ±8.51	0 (AD) to 75 (CN)

Table S2: Tuned hyperparameters for different baseline methods

LR	Alpha
SVM	C, gamma, kernel
RF	Node estimators, node depth, min. samples split, min. samples at leaf, bootstrap
NN	Batch size, epoch, L2, drop out rate, hidden layer size, number of layers, activation

Table S3: Gene-level mapping of SNPs, DNA methylation CpG sites and gene expression probes identified by the HINN model.

Gene	Gene Expression Probes	DNA Methylation Sites	Associated SNPs
RCHY1	11719570_at, 11719571_a_at	cg16542283	4:76838909, rs72655514
AGPAT1	11730933_a_at, 11750187_a_at, 11750188_x_at, 11751668_a_at	cg03718284	6:31574685
RNF5	11757623_s_at, 11762163_at	cg03718284	6:31574685
C19orf62	11716039_a_at, 11748858_a_at, 11761811_at	cg07697134	19:16412710, 19:16412713
PNPLA2	11730221_at, 11730222_x_at, 11730223_a_at	cg12129309	11:1127827, 11:1421705, 11:1449993, 11:1620897, 11:383205, rs4963152
BUB1B	11720647_a_at	cg11331988, cg24451981	rs142664254, rs146462082, rs142664254, rs146462082
ATP6V1C1	11722181_a_at, 11722182_a_at, 11722183_s_at, 11745272_a_at, 11758863_at	cg08793540	8:103465626, 8:103478333, 8:103478335, 8:103478339, rs137993565, rs138982787, rs145145835, rs146795324, rs146905769, rs147749199, rs147759363, rs148729689, rs148985239, rs181936096, rs186121690, rs192189961
CARD11	11723142_at	cg26937500	7:2695718

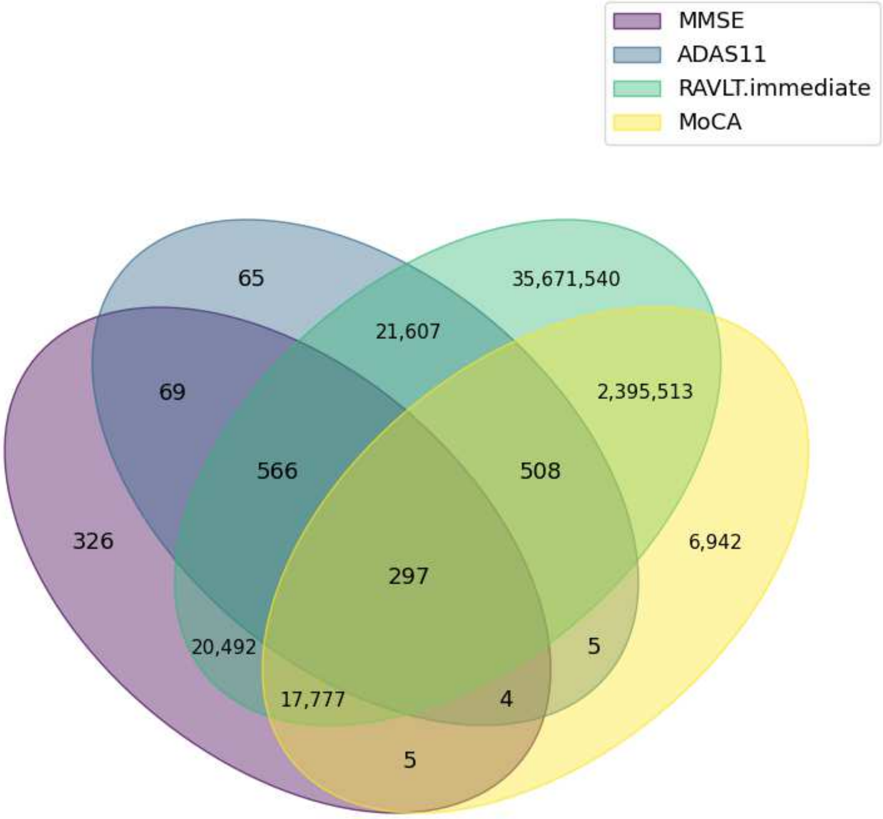

Figure S1: Venn diagram of GWAS identified significant SNPs associated with each cognitive measurement.

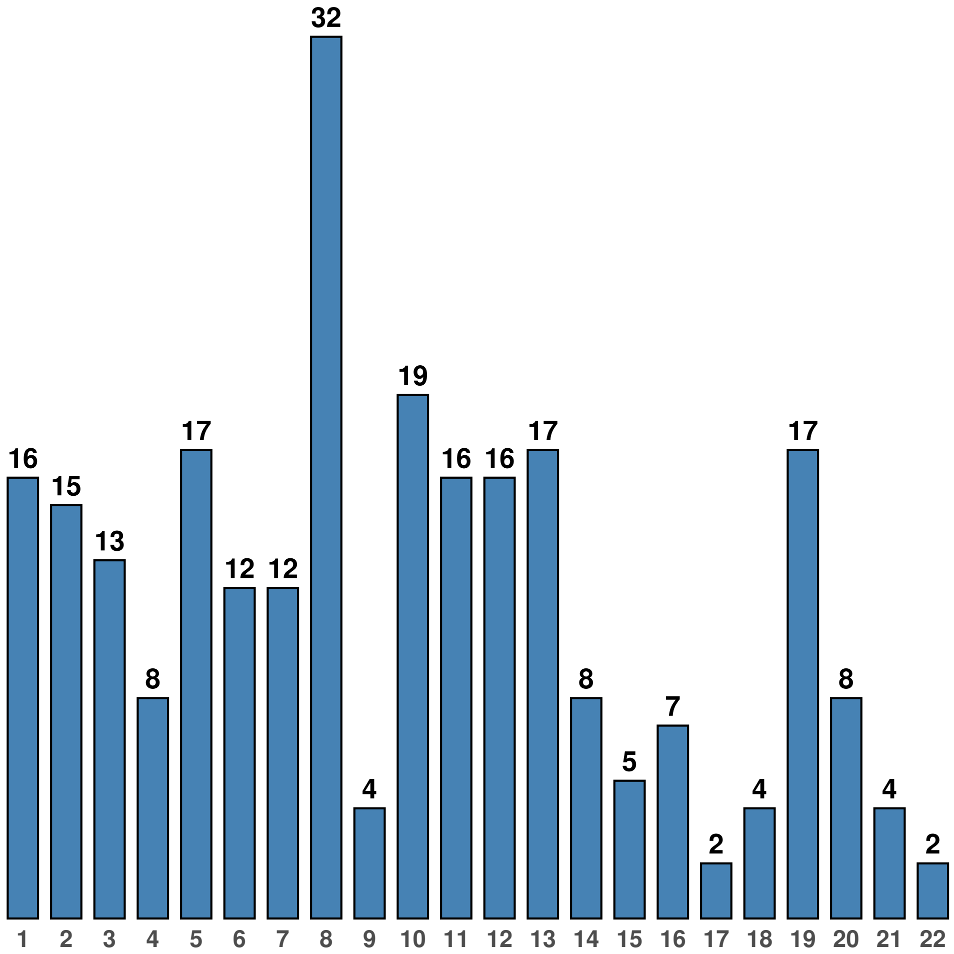

Figure S2: Number of significant SNPs that had associated CpG sites across each chromosome highlighting genomic regions commonly implicated in cognitive variation.

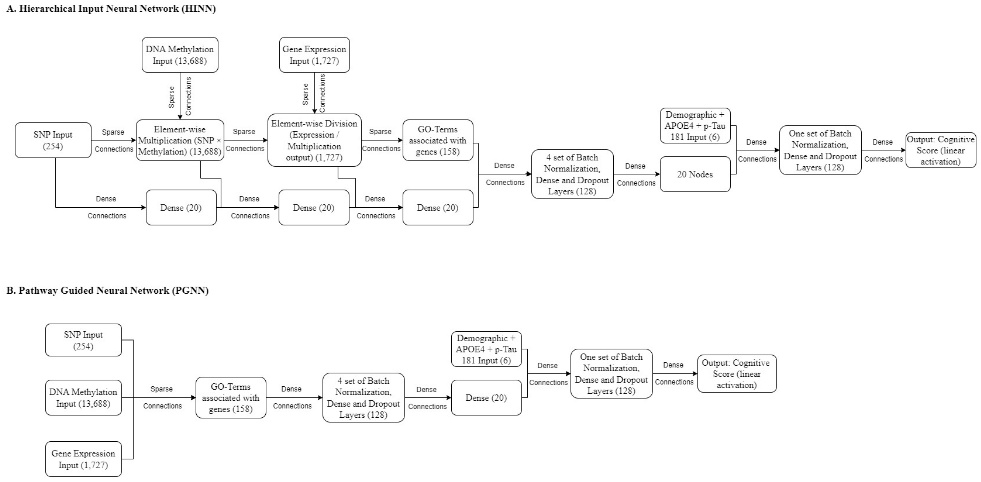

Figure S3: Model Architecture. (A) The Hierarchical Input Neural Network (HINN) architecture integrates three omics data types—SNP genotypes, DNA methylation profiles, and gene expression levels—along with demographic and biomarker features to predict cognitive scores. The model incorporates biologically informed transformations, including element-wise operations and GO term annotations, to enhance interpretability and biological relevance. (B) The Pathway-Guided Neural Network (PGNN) architecture connects each omics input directly to GO terms, leveraging structured biological knowledge for feature aggregation. The downstream architecture, including the dense layers and cognitive score prediction, mirrors that of the HINN model.

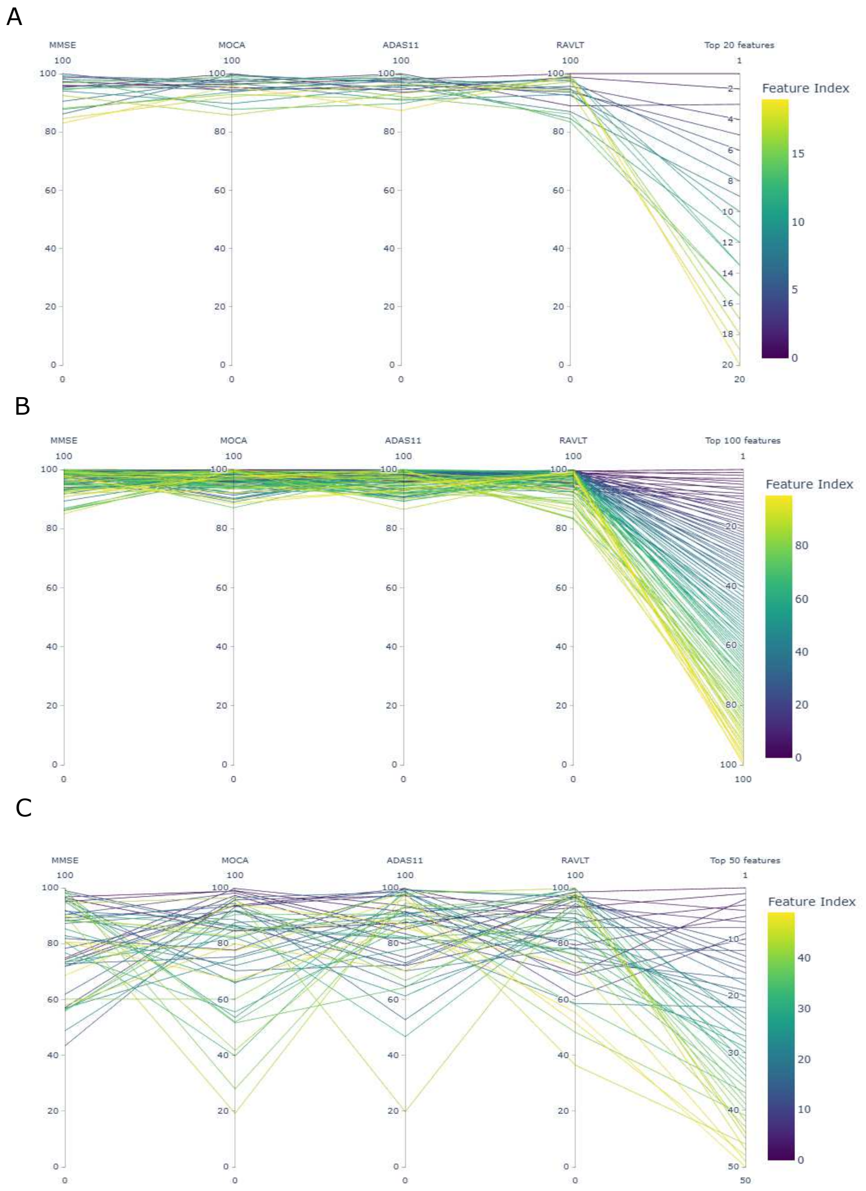

Figure S4: Top common features based on their importance across different cognitive tests (A) Top 20 SNP features (B) Top 100 DNA methylation features (C) Top 50 gene expression features.
